# Effect of Preservative Pretreatment on the Biological Durability of Corn Straw Fiber/HDPE Composites

**DOI:** 10.3390/ma10070789

**Published:** 2017-07-12

**Authors:** Lihui Xuan, Dongxue Hui, Wanli Cheng, Andrew H. H. Wong, Guangping Han, Wei Khong Tan, Carlson A. D. Tawi

**Affiliations:** 1 Key Laboratory of Bio-Based Material Science and Technology (Ministry of Education), Northeast Forestry University, Harbin 150040, China; leeh91@hotmail.com (L.X.); Dongxue_zylc@126.com (D.H.); nefucwl@nefu.edu.cn (W.C.); 2 Faculty of Resource Science & Technology, Universiti Malaysia Sarawak, Kota Samarahan, Sarawak 94300, Malaysia; ahhwong@unimas.my (A.H.H.W.); weikhong90@hotmail.com (W.K.T.); carlsontawi@gmail.com (C.A.D.T.)

**Keywords:** corn stalk fiber, high-density polyethylene, alkaline copper quaternary, zinc borate, termite, mold, wood-decay fungi

## Abstract

The effects of alkaline copper quaternary (ACQ) and zinc borate (ZB) on the resistance of corn stalk fiber (CSF)-reinforced high-density polyethylene (HDPE) composites to biodegradation were examined. Both biocides could inhibit termites, mold fungi, and wood-decay fungi, even at high CSF formulations (i.e., 60%). Additionally, ACQ enhanced the resistance of the composite materials to certain biotic stresses better than ZB. The CSF/HDPE composites treated with ACQ at the 3.0% level exhibited a superior performance against termites, white rot fungi, and brown rot fungi. ACQ treatment at the 1% level was optimal for inhibiting soft rot fungi. Furthermore, mold growth was not observed on ACQ-treated CSF/HDPE samples. The untreated CSF/HDPE composites were more susceptible to mold infections and decay than the untreated poplar/HDPE composites, likely because of an incomplete removal of the pith. The chemical features of the corn stalk may also have influenced these differences, but this possibility will need to be explored in future investigations. Furthermore, the CSF component of CSF/HDPE composites is highly susceptible to fungal attacks, with the soft rot fungus inducing the largest mass losses, followed by the white rot fungus, and then the brown rot fungus.

## 1. Introduction

Corn is an important agricultural crop in northeastern China, and its annual production in the Heilongjiang province of 33.43 million tons accounts for more than 14.5% of the total yield in China [1]. Corn stalks, which are the agricultural residues of corn crops, comprise more than half of the crop yield. With an annual production of nearly 207 million tons, corn stalks have been burned in large quantities on farmlands, producing seasonal smog and aggravating the air pollution in China [2]. Agricultural fibers, such as corn stalk [3,4,5], sunflower stalk [4] and oilseed stalk [5], contain an abundance of lignocellulosic fibers, making them a potential raw material for manufacturing composite materials.

Because of a shortage of available wood resources and the detrimental environmental impact of burning agricultural residues, studies have been conducted over the last few decades regarding agricultural fiber-based composites in China [6,7]. One viable solution involves using agricultural residues as raw materials for making agricultural fiber-plastic composites (AFPCs). At similar densities, AFPCs are comparable to wood-plastic composites (WPCs) regarding their physical and mechanical properties [8,9,10]. The potential benefits associated with recycling agricultural residues to produce AFPCs include the reduction in carbon emissions and preservation of wood resources. Meanwhile, AFPCs also can avoid the white pollution created by non-biodegradable plastic.

AFPCs have prevailed as outdoor building materials, such as dock fences, pavilions, and road signs, which are exposed to cyclical wet and dry outdoor environments. Though HDPE and Maleated polyethylene (MAPE) materials are immune to biological attacks, the AFPCs are susceptible to bio-deterioration under prolonged exposures to high humidity or water, which can severely affect the economic value and utility of the products. In fact, the agricultural fibers in AFPCs are susceptible to wood-decay fungi, termites, and mold fungi [4,5]. A previous study revealed that sugars and starches are more abundant in corn stalk than in wood [11]. Therefore, appropriate chemical treatments are required to protect AFPCs made from corn stalk fiber (CSF) from biotic stresses. Various preservatives and treatments have been used to enhance the biological performance of WPCs. Samples containing recycled chromed copper arsenate-treated wood exhibit improved biological durability and photo protection properties [12]. Additionally, WPCs containing alkaline copper quaternary (ACQ)-treated or micronized copper quaternary-treated wood show less surface-level wood-decay fungal growth and mass loss due to termites than WPCs prepared using untreated wood [13]. According to decay and termite tests, increasing the content of zinc borate (ZB) to the 1% (w/w) retention level significantly decreases the mass loss rates of WPCs [14].

ACQ is one of the copper amine-based preservatives, which has the advantages of easy treating practices, low volatile organic compound emissions, and improved anti-biological properties. With the proven efficacy of ACQ treatments of wood products, it is hypothesized that using ACQ-treated agricultural fibers as raw materials during the production of AFPCs will decrease the susceptibility of the final products to biological degradation and photo degradation. As one of the preservatives used to minimize the damage caused by insects and microbes, ACQ can extend the service life of decking materials [15]. ZB was primarily used as a preservative and insecticide, containing isolated polyborate anions, and the principal excellences were their relatively lower toxicity and uniform color of treatment. In other studies, ZB was combined with wood flakes during the production of composites to protect the final products from termites and decay [6,16]. Additionally, there is a detailed report published on ZB treatments and their performance [17]. However, attempts to study the effect of ACQ- or ZB- modified CSF on the anti-biological properties of AFPCs are relatively rare.

The objective of this study was to investigate the feasibility of using ACQ and ZB as preservatives during the production of AFPCs from CSF. The effects of various ACQ and ZB treatments on the performance of composites against termites, mold fungi, and wood-decay fungi were evaluated.

## 2. Materials and Methods

### 2.1. Raw Materials and Preparation

Corn stalks were collected from farmland in Harbin, China. After removing the inner pith, the corn stalks were granulated using a laboratory mill to the size required to pass through a 60-mesh screen. The average length of CSF was 1 mm, and the average diameter was 0.1 mm. The ground material was dried to a moisture content of about 3% prior to use. We used commercially available ACQ and ZB as preservatives (Star Wyatt Wood Preserving Co. Ltd., Guangzhou, China). Preservative-treated CSF was prepared by vacuum-impregnating samples with ACQ or ZB. High-density polyethylene (HDPE) (density: 954 kg/m^3^ and flow index: 0.7 × 10^−4^ kg/min at 190 °C) was used as the matrix (Quan Ji Plasticization Co. Ltd., Suzhou, China). MAPE (Epolene CMG9804 with a grafting rate of 0.9%) was used as the coupling agent (Aladdin Reagent, Shanghai, China). Paraffin was used as lubricant (Aladdin Reagent, Shanghai, China).

### 2.2. Blend Design and Sample Fabrication

Table 1 presents the design of various blends. Composite samples were prepared in the Engineering Composite Laboratory of Northeast Forestry University using the SJSH30/SJ45 twin-screw extruder with a 4 mm × 40 mm die manufactured by the Nanjing Rubber Machinery Plant in Jiangsu, China. The blending temperature profile was set at 145, 155, 160, 165, 165, 155, 150, and 145 °C from the feeding zone to the die. Preservative-treated CSF, HDPE, and other processing components (i.e., 2% maleated polyethylene and 1% paraffin) were added to the extruder and thoroughly mixed. The CSF/HDPE profile, which was about 4 mm thick, was cut into 100 mm × 40 mm blocks for the termite test, 50 mm × 25 mm blocks for the mold test, and 30 mm × 30 mm blocks for the wood-decay fungi test. The moisture content was determined after blocks were air-cooled, but before the termite and mold tests were conducted.

### 2.3. Biological Tests

#### 2.3.1. Subterranean Termite Field Test

Three replicated test blocks (100 mm × 40 mm) of each type of a treated or control CSF/HDPE sample were oven-dried at 105 °C for 48 h for wood moisture content measurements. The moisture content data were then used to estimate the oven-dried masses of six replicated test blocks for an aboveground termite field test. Blocks cut from poplar (*Populus* sp.) wood, which is susceptible to termites, were used as reference material to be tested under the same conditions. The aboveground subterranean termite field test protocol (i.e., protected from external weather conditions with moderate humidity inside the container) used in this study was designed according to Wong [18], adapted from the method of Creffield [19]. The method simulates the Malaysian Hazard Class H2 exposure conditions for treated and untreated wood used in Malaysia (i.e., aboveground indoors and subjected to only termites and wood-boring beetles) [20]. By limiting the moisture entering the termite test containers, we were able to prevent fungi from colonizing specimens, particularly mold fungi, which would have otherwise inhibited termite attacks.

The termite field test was conducted at a well-drained forested field at Universiti Malaysia Sarawak (Unimas), Kota Samarahan, Sarawak, Malaysia. The field site was infested with *Coptotermes curvignathus*. The termite test container consisted of a rectangular metal container (30 cm × 30 cm × 40 (H) cm) that was divided into a top and bottom half. Mid-height inside the container (i.e., 20 cm height), untreated and preservative-treated CSF/HDPE samples were randomly positioned 20 cm aboveground on a Nylex™ (Sarawak, Malaysia) mesh supported by a 20 cm tall aluminum stand placed on the ground inside each container. The position-mapped test specimens resting on a mesh 20 cm aboveground were supplemented with additional termite-susceptible woody bait materials. Untreated poplar wood blocks, pinewood residue, rubberwood residue, and corrugated cardboard waste were placed alongside treated (test) specimens to lure subterranean termites to the test specimens. In addition to the test specimens, the top half of each container was filled almost to capacity with the termite-susceptible materials such that the test specimens were concealed within the bait material.

**Figure 1:**
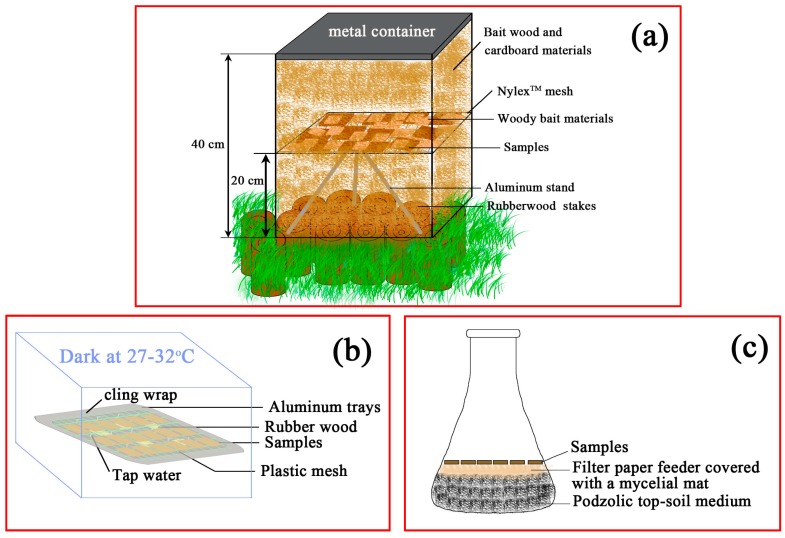
Schematic drawing of biological test methods. (**a**) Subterranean termite field test; (**b**) Laboratory mold test; (**c**) Decay resistance test.

The bottom half of the containers (20 cm height) served as a conduit for the subterranean termites to access the test specimens. The bottom of the unsealed containers sat on topsoil with grass cover removed underneath. The conduit resembled a bait station containing a few pinewood stakes pegged into the ground within the container. The bait wood and cardboard materials added to the upper half of the containers were also used to fill the bottom half. Additional stakes were pegged along the exterior of the container to attract termites to the vicinity of the containers. Metal lids were used to secure the containers to prevent rainwater and light from entering for the duration of the field test. The termite test assembly was inspected weekly by carefully removing the lids and checking for evidence of termites foraging on the upper layer of the bait material. This would indicate that the termites had entered the containers from the ground directly below and moved upward past the section containing the test specimens. The schematic drawing is presented in Figure 1a.

The termite test was completed after 12 weeks when heavy termite cartons (i.e., mixture of faecal matter and wood fragments) were detected in the mixed bait material at the top of the containers. Damp test specimens were removed from the containers, carefully cleaned by brushing off any soil and carton debris, and oven dried at 105 °C for 48 h. Specimens were visually rated using the American Wood Preservers’ Association 10-point termite rating scale of 10 (sound), 9.5 (trace, surface nibbles permitted), and 9 (slight attack, up to 3% cross-sectional area affected), down to 0 (destroyed, >75% of cross-sectional area affected). We then calculated the mass loss (in mg and % mass/mass) of the CSF/HDPE test blocks.

#### 2.3.2. Laboratory Mold Test

Test blocks (50 mm × 25 mm) of CSF/HDPE, poplar, and mold-susceptible rubberwood (Heveabrasiliensis) were used for the laboratory mold growth test (*n* = 6). Aluminum trays were half-filled with tap water and then covered with a plastic mesh support. The randomly positioned air-dried test specimens were placed above the water level, but were still exposed to high humidity (i.e., 80–94% relative humidity). The samples were incubated in darkness at 27–29 °C. Mold fungus-infected rubberwood sapwood bait blocks were immersed in the tap water below the mesh support to provide a mixed natural inoculum for infecting the CSF/HDPE, poplar, and rubberwood test specimens. Three such tray assemblies were prepared, with cups of water placed in the aluminum trays to maintain the humid conditions conducive for mold infections of at least the susceptible rubberwood and poplar blocks. The schematic drawing is presented in Figure 1b. The oven-dried (105 °C) masses of the samples were estimated based on the moisture content measurements. The samples were visually rated and weighed daily, beginning the day after the incubation was initiated. The incubation was stopped when the top and bottom surfaces of all rubberwood and poplar specimens had an infection rating of 6 (median: 88% surface coverage). Thus, the test was terminated after a 30-day incubation period. By the end of the test period, the moisture contents of the various CSF/HDPE samples had increased from 0.2–4% to 4–25.4%. The moisture contents of the rubberwood and poplar samples had increased from 9.4–16.5% to 37.9–51.8%. The criteria used to visually rate the severity of the mold infections, pooling the top and bottom faces of each CSF/HDPE specimen, are listed in Table 2. A 6-point mold growth visual rating scale was adopted (rating 0: no mold growth; rating 6: mold growth coverage over the pooled top-bottom surfaces between 75 and 100% and known as 88% median mold growth severity). Of all the ratings, there is at least one representing a range of mold growth coverage on the pooled top and bottom surfaces of the specimens. Based on the visually determined infection range, a median mold infection percentage was assigned as described by Wong et al. [21]. For commercial production, any specimen with mold growth coverage >50% is considered to have failed the test (i.e., a rating of at least 5), even though it is not completely covered with mold.

#### 2.3.3. Decay Resistance Test

Decay resistance tests for CSF/HDPE samples with different concentrations of preservatives, species, and CSF content were conducted according to the general laboratory procedure of ASTM D2017-81 (ASTM International, Philadelphia, PA, USA) [22] against Basidiomycetes and the unsterile soil burial method of Wong [23] against soft rot fungi. The controls for this test consisted of poplar sapwood and different formulations of poplar/HDPE composites, while rubberwood blocks served as sacrificial inspections to monitor the progress of all three decay tests and to decide on the duration of the soft rot test. Samples were inoculated with eithera white rot fungus (*Pycnoporus sanguineus*), brown rot fungus (*Gloeophyllum trabeum*), or soft rot fungi (*Chaetomium globosum* mixed with an unknown microbial population in unsterile soil). Test samples were exposed to *P. sanguineus* and *G. trabeum* for 16 weeks, and the *C. globosum* unsterile soil soft rot mixed inocula for 52 weeks when for these tests sacrificial rubberwood blocks would have recorded at least 60% mass loss. Each treatment comprised six replicates for each wood decay type being tested. The generated data for the six samples underwent a single-factor variance analysis to produce the final test results.

For the ASTM soil-jar decay test [22], petri dishes of 2% malt agar medium were aseptically inoculated with white rot and brown rot fungi, and were incubated at 28 ± 2 °C for a week. The fungal cultures were then aseptically transferred to the autoclaved (122 °C, 120 KPa, 30 min) soil-jar assembly, where each 375-mL soda-glass jar contained 150-mL podzolic top-soil medium (soil moisture content: 130% of water-holding capacity) with 2% malt-enriched Whatman No. 1 filter paperfeeders placed on top of the soil. For the soft-rot unsterile soil burial test using similar jars [23], the non-autoclaved soil-jar assembly contained unsterile podzolic soil (i.e., contaminated with unknown ascomycete/deuteromyceteinocula and at 130% of soil water holding capacity), in which soft rot fungal inoculum was placed on 2% malt-enriched filter paper laid on the soil surface within the jar that held the buried unsterile test block.

The test blocks (30 mm × 30 mm × 4 mm) were oven-dried at 40 °C to reach a constant weight (W1), which was the initial weight before the decay test. The test blocks meant for the *Basidiomycete* tests were grouped together and fumigated with propylene oxide for 24 h, and each block was then aseptically placed in a soil-jar assembly on top of the filter paper feeder covered with a mycelial mat. Test blocks meant for the soft rot unsterile soil-burial test were not fumigated, but each block per jar was buried 2-cm in the unsterile soil and then inoculated with *C. globosum* on to 2% malt-soaked filter paper resting at the top of the soil. The *Basidiomycete* decay test soil-jar assembly was incubated at 27 °C and 80% relative humidity for approximately 16 weeks, by which time additional sacrificial rubberwood inspection blocks reached 50–60% decay mass loss. For the soft rot test, incubated under similar conditions, it took 52 weeks for sacrificial rubberwood blocks to reach such high mass losses. At the end of these three decay tests, all samples were then removed from these soil-jars and carefully cleaned to remove the mycelia or soil debris from the block surface. The blocks were stored at room temperature for 24 h and then oven-dried at 40 °C to a constant weight (W2). The mass loss (%) was calculated based on the difference in the oven-dried weights of each specimen before and after the decay test: Weight loss (%) = [(W1 − W2)/W1] × 100%. The schematic drawing is presented in Figure 1c.

### 2.4. Scanning Electron Microscope

A scanning electron microscope (SEM, QUANTA-200, FEI, Hillsboro, OR, USA) was used to obtain microphotographs of CSF/HDPE composites. The samples were coated with a layer of gold-palladium before being observed with SEM at an accelerating voltage of 12.5 kV.

## 3. Results and Discussion

### 3.1. Termite Resistance

Termites are distributed in tropical and subtropical regions and like to nest in areas close to water. The required nutrients of termites are derived from plant cellulose, lignin, and their products. The termite test data are summarized in Table 3. The extent of the termite damage was based on the AWPA 0–10 rating scale (rating of 0: complete destruction of the sample; rating of 10: no evidence of termite attack). The ratings for the CSF/HDPE samples with preservative (ACQ or ZB) retentions of 1.0%, 2.0%, or 3.0% were all above 9.0, proving the better resistant performance of preservatives to termites. In contrast, the ratings for untreated CSF and pure poplar were 8.8 and 0.0, respectively, which indicated HDPE could protect CSF from termite erosion to some extent. Of the samples from two groups with the same CSF formulations (50/50 or 60/40), the samples with ACQ-treated CSF had a higher rating than the samples with ZB-treated CSF. This implies that ACQ enhances termite resistance more than ZB. As for the effect of preservative content on the visual rating, no significant changes were recorded with different preservative-incorporated formulations, showing that the preservative content level had little effect on the visual rating in this experiment.

**Figure 2:**
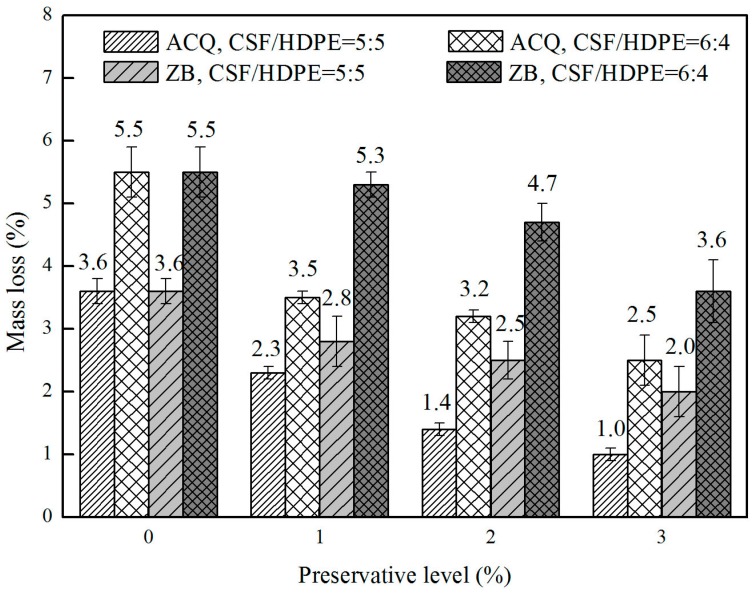
Mean mass loss of different composites on the termite resistance test.

The average mass losses for the ACQ-treated CSF/HDPE, ZB-treated CSF/HDPE, and untreated CSF/HDPE with different formulations are provided in Figure 2. The ACQ-treated and ZB-treated CSF/HDPE samples retained more mass than the untreated CSF/HDPE sample, indicating that the preservative-treated CSF materials enhanced the termite resistance of the polymer composites. In samples with the same formulation, increasing preservative concentrations were associated with decreasing mass losses. The mass loss decreased from 3.6% (control: no ACQ) to 1% (ACQ level: 3%) for the ACQ incorporated in 50/50 formulations, and from 5.5% (control: no ACQ) to 2.5% (ACQ level: 3%) for the ACQ incorporated in 60/40 formulations. These results indicate that the ACQ treatment affected the ability of the termites to damage the composite materials. Regarding the ZB treatments, the mass loss decreased from 3.6% (control: no ZB) to 2% (ZB level: 3%) for the ZB incorporated in 50/50 formulations, and from 5.5% (control: no ZB) to 3.6% (ZB level: 3%) for the ZB incorporated in 60/40 formulations. Samples with ACQ-treated CSF had a lower mass loss than samples with ZB-treated CSF. This implies that ACQ enhances termite resistance more than ZB.

In general, a higher mass loss was observed with increasing CSF content, likely because the termites were able to feed on more CSF material in this no-choice test. These findings are consistent with the results of a previous study [14]. As expected, high encapsulation rate formulations (50/50) produced lower mass losses. Regarding high CSF content formulations (60/40), the mass losses were high after a 12-week above-ground field exposure to *C. curvignathus*. This result indicated that the CSF material in the CSF/HDPE composite was heavily damaged by termites. With increasing ACQ retention levels, the termite resistance initially increased rapidly, but then increased more slowly for both formulations. Termite resistance increased 2.6- and 1.2-fold at an ACQ level of 3% for the 50/50 and 60/40 formulations, respectively. Shang et al. [13] also reported that termite resistance increased by approximately three-fold at an ACQ level of 4% for the 40/60 (wood/plastic) formulation. The increase in termite resistance was smaller for the ZB treatment than for the ACQ treatment. A previous study confirmed that ZB-incorporated WPC specimens had considerably lower mass losses than untreated specimens. The mass losses due to termite feeding generally increased by more than two-fold when the particle content increased from 50% to 70% [24]. This is likely because the particles are biodegradable, which is an important factor to consider when analyzing the degradation of such composites by termites.

### 3.2. Mold Resistance

In the short term, mold fungi may be more important for WPCs than wood-decay fungi because they develop more quickly on the WPCs [25]. Data regarding the timing of the mold infection and the median mold growth rate are summarized in Table 4. At the end of the 30-day test period, there was no detectable mold growth in the ACQ-treated CSF/HDPE composites, even for the 60/40 formulation. This result implies that ACQ provides the composites with some protection from infections by mold fungi. In contrast, the timing of the initial mold attack was about the same for the ZB-treated and untreated composites. In conclusion, ZB had no significant effect in the early stages of mold growth. However, the median mold growth of ZB treated samples was lower than the control groups in the end stage, indicating that ZB treatment slightly enhanced mold resistance. Compared with untreated poplar/HDPE composites, more molds grew on the untreated CSF/HDPE composites at the end of the test period. This difference is likely due to the chemical features of the corn stalk. A previous study revealed that corn stalk contains more sugars and starches than wood [11]. Therefore, as long as the sugars and starches are present, CSF/HDPE will likely remain more susceptible to mold fungi than the composites lacking CSF. Moreover, it can be attributed to the different structures between CSF and poplar, such as the specific surface area, crystallinity, and aggregation. Han et al. [6] also reported that the susceptibility of the oriented structural straw board to mold fungi was higher than that of pine wood.

**Figure 3:**
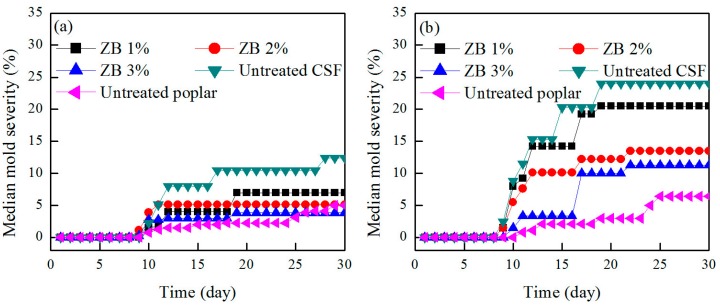
Median mold severity of ZB-treated CSF/HDPE composites compared with untreated CSF and untreated poplar. (**a**) CSF/HDPE = 50/50; (**b**) CSF/HDPE = 60/40.

The median mold infection severities for the ZB-treated CSF/HDPE, untreated CSF/HDPE, and untreated poplar/HDPE with different formulations are presented in Figure 3. The severity of the mold infection was lower for the ZB-treated CSF/HDPE than for the untreated CSF/HDPE, indicating the preservative treatment can enhance the mold resistance of polymer composites. Increasing ZB levels were associated with a decreasing mold infection severity for the same formulations. Additionally, mold infections were more severe in untreated CSF/HDPE than in untreated poplar/HDPE samples, indicating that mold can grow faster on CSF than on solid wood. However, with increasing ZB levels, the severity of the mold infection on ZB-treated CSF/HDPE approached the severity of the mold infections on poplar/HDPE composites.

When the CSF content increased to 60%, the median mold infection severity increased from 12.4% to 24%, which corresponded to a rating increase from 2 to 3, depending on the formulation type. The superior mold resistance of the 50/50 composites is probably because the lower CSF content in the composites ensures that it is well encapsulated and protected by the plastic. These findings are consistent with previous observations, which suggested that there is a linear correlation between wood content and mold infection severity. This may be attributed to an increase of wood not encapsulated by the polymer matrix [26]. Our results also revealed that the mold resistance of CSF/HDPE composites varied with the retention of ZB. The mold infection severity significantly decreased as the ZB levels increased for both formulations. For the 60/40 formulation, the addition of ZB to the 3% level significantly decreased the mold infection severity from 24% to 11.25%, with a decline in the rating from 3 to 2. These results imply that the biocide used inhibits mold fungus activity, even at high CSF formulations.

The curvilinear trend of all samples was similar, and the median mold severity was increased with an increasing test time. As can be seen, the mold increment was apparent within the first 20 days, and then gradually became slower. This is because the limited CSF was on the surface of composites, so the mold growth rate decreased with the reduced CSF content.

### 3.3. Resistance to Wood-Decay Fungi

#### 3.3.1. Resistance to White Rot Fungus

**Figure 4:**
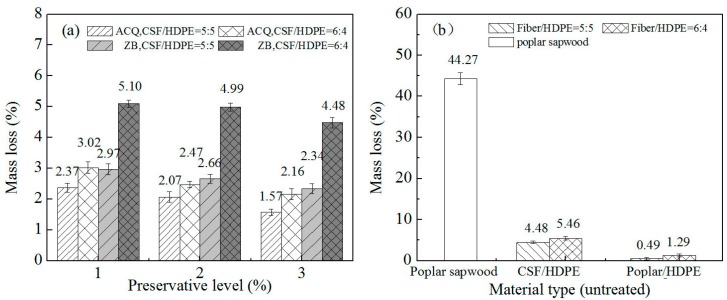
Mean mass loss of different composites on the white-rot (*Pycnoporous sanguineus*) decay resistance test. (**a**) CSF/HDPE composites treated by ACQ or ZB; (**b**) untreated control groups.

The weight loss results for the CSF/HDPE and control specimens after analyzing the resistance to the white-rot fungus are presented in Figure 4. The decay resistance of CSF/HDPE varied depending on the preservative treatment and the CSF/HDPE formulations. The ACQ-treated or ZB-treated CSF/HDPE samples retained more mass than the untreated CSF/HDPE, indicating that the preservative treatments enhanced the resistance to the white rot fungus. Additionally, increases in preservative abundance were accompanied by decreases in mass loss for the same formulations. The mass loss decreased from 2.37% (ACQ level: 1%) to 1.57% (ACQ level: 3%) for the ACQ incorporated in 50/50 formulations, and from 3.02% (ACQ level: 1%) to 2.16% (ACQ level: 3%) for the ACQ incorporated in 60/40 formulations. The ACQ treatment clearly increased the white rot resistance of the composites. For the ZB treatments, the mass loss decreased from 2.97% (ZB level: 1%) to 2.34% (ZB level: 3%) for the ZB incorporated in 50/50 formulations, and from 5.51% (ZB level: 1%) to 4.48% (ZB level: 3%) for the ZB incorporated in 60/40 formulations. Thus, the ZB treatment had little effect on white rot resistance. The samples with ACQ-treated CSF were more resistant to the white rot fungus than the samples with ZB-treated CSF, even at high CSF formulations. These results indicate that ACQ can enhance decay resistance more than ZB. Moreover, the untreated CSF/HDPE composites with a lower CSF content (50/50) had a relatively low mean mass loss (4.48%). When the CSF content increased to 60%, the mass loss also increased (5.46%). This implies that the 50/50 formulations provided better encapsulation of the CSF by the HDPE matrix. These observations are consistent with those of a previous study [14].

In general, the mass loss caused by *P. sanguineus* was uniformly low for all composites tested. However, the mass loss for the untreated poplar sapwood reference materials was 44.27%, indicating that the CSF/HDPE composites were far more decay-resistant than solid wood, even at high CSF formulations. This is likely because the weight loss in WPCs is only based on the fiber filler, and the WPC fibers are a possible food source for fungi [27]. *P. sanguineus* induced a significant weight loss in untreated CSF/HDPE composites, but not in poplar/HDPE. The weight loss in untreated CSF/HDPE composites was 4.48% in the 50/50 formulations and 5.46% in the 60/40 formulations. However, the fact that the *P. sanguineus* induced weight loss was only 0.49% (50/50) and 1.29% (60/40) for poplar/HDPE, suggests that CSF is more susceptible to the white rot fungus than the poplar fibers.

#### 3.3.2. Resistance to Brown Rot Fungus

**Figure 5:**
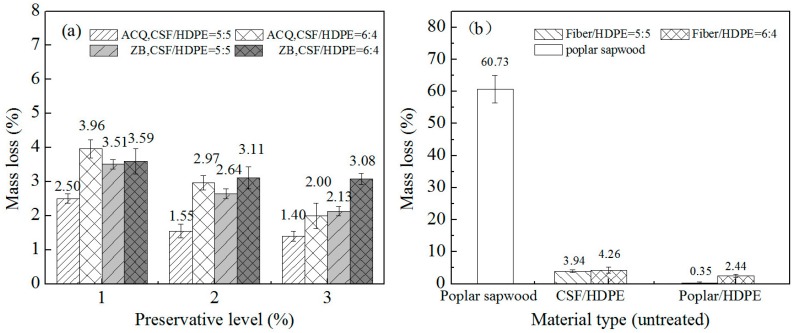
Mean mass loss of different composites on the brown-rot (*Gloeophyllum trabeum*) decay resistance test. (**a**) CSF/HDPE composites treated by ACQ or ZB; (**b**) untreated control groups.

A summary of the mass losses for the CSF/HDPE composites with different formulations and control specimens is provided in Figure 5. The mass loss due to the brown rot fungus (*G. trabeum*) was smaller than that caused by the white rot fungus in all untreated CSF/HDPE samples, regardless of the formulation. These results are in agreement with those described by Verhey et al. [28]. This diversity between the effects of the two fungi may be because of differences in the CSF components that are attacked. The white rot fungus targeted lignin, while the brown rot fungus mainly attacked cell wall carbohydrates. Additionally, there were no significant differences between the resistance of the two fungi for preservative-treated samples, indicating that ACQ and ZB can inhibit both fungi. As with the white rot fungus, the mass loss caused by *G. trabeum* was consistently lower for all tested composites than for the poplar control specimens (70.73%), indicating the existence of an invasive decay environment and active fungal viability in poplar. These findings are consistent with the results of a previous study by Simonsen [29]. Meanwhile, the weight loss was greater for the untreated CSF/HDPE composites than for the untreated poplar/HDPE composites for the same formulations. A similar observation was reported by Feng [30], suggesting that there are considerable mass loss differences among HDPE/wood and HDPE/bamboo composites. This provides additional evidence that the chemical features of fibers can influence decay resistance. Moreover, the mass loss of 60/40 formulations were uniformly high for all tested preservative levels, while the mass loss decreased for the 50/50 formulations. Therefore, the CSF/HDPE component ratio is an important factor influencing the resistance to the brown rot fungus.

The untreated composite samples exposed to *G. trabeum* lost considerably more weight than the samples treated with ACQ or ZB. Additionally, increasing preservative concentrations resulted in a decreasing mass loss for the same formulations (Figure 5). For example, increasing the ACQ level from 1% to 3% decreased the mass loss of the composite materials from 2.5% to 1.4% (50/50) and from 3.96% to 2.00% (60/40). The most obvious composite mass loss decreases occurred at low ACQ concentrations. As the preservative concentration increased, the mass loss decreases became smaller. In contrast, there were no significant differences among the ZB treatments for the 60/40 formulations. The mass losses were clearly larger for the ZB-treated CSF/HDPE composites than for the ACQ-treated specimens, indicating that ACQ contributed to brown rot resistance more than ZB.

#### 3.3.3. Resistance to Soft Rot Fungus

**Figure 6:**
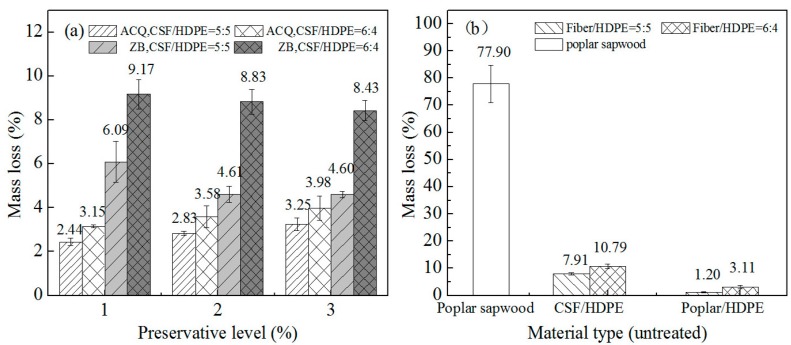
Mean mass loss of different composites on the soft-rot (*unsterile soil incorporating Chaetomium globosum*) decay resistance test. (**a**) CSF/HDPE composites treated by ACQ or ZB; (**b**) untreated control groups.

The average soft rot fungus-induced mass losses in composite materials treated with ACQ or ZB are provided in Figure 6. Compared with the effects of the white rot and brown rot fungi, the soft rot fungus (*C. globosum*) caused greater mass losses in all untreated CSF/HDPE composites at different formulations. This suggests that CSF/HDPE composites are highly susceptible to soft rot fungal infections. Regarding the influence of CSF content, a lower soft-rot resistance was observed with an increasing CSF content. This was the expected result because CSF can provide an environment conducive for composite decay. Thus, mass losses were higher for the 60/40 CSF formulation than for the 50/50 formulation. These findings imply that the CSF content is an important factor affecting the degradation behavior of CSF/HDPE composites infected by the mixed soft rot fungal inocula. Our data is in agreement with the results described in earlier publications [13,24]. As expected, there were significant differences in the mass loss between CSF/HDPE and the poplar wood control sample. Mass losses were high for pure poplar, verifying that the CSF in composites can be attacked by wood-decay fungi. Compared with the untreated poplar/HDPE composites, untreated CSF/HDPE composites were less resistant to the mixed soft rot fungal inocula.

The ACQ and ZB-treated composites retained more weight than the untreated samples, indicating that the preservatives may be useful for enhancing soft-rot resistance. A previous study concluded that the incorporation of ZB in composite materials can significantly decrease mass losses, even at high fiber component formulations [14]. With increasing ZB levels, the mass losses due to soft rot decay decreased in the same formulations. The initial considerable decrease in mass loss became smaller as the ZB level increased. The mass loss decreased from 7.91% (control: no ZB) to 4.61% (ZB level: 3%) for the ZB incorporated in 50/50 formulations, and from 10.7% (control: no ZB) to 8.43% (ZB level: 3%) for the ZB incorporated in 60/40 formulations. Although ACQ treatments decreased mass losses, there were no major differences among the ACQ levels. The mass losses changed from 2.44% (ACQ level: 1%) to 3.02% (ACQ level: 3%) for the ACQ incorporated in 50/50 formulations, and from 3.15% (ACQ level: 1%) to 4.12% (ACQ level: 3%) for the ACQ incorporated in 60/40 formulations. Furthermore, mass losses were lower for samples with ACQ-treated CSF than for samples with ZB-treated CSF. This suggests that ACQ provides a greater resistance to *C. globosum*/unsterile soil mixture than ZB.

#### 3.3.4. Scanning Electron Microscopy Analysis

**Figure 7:**
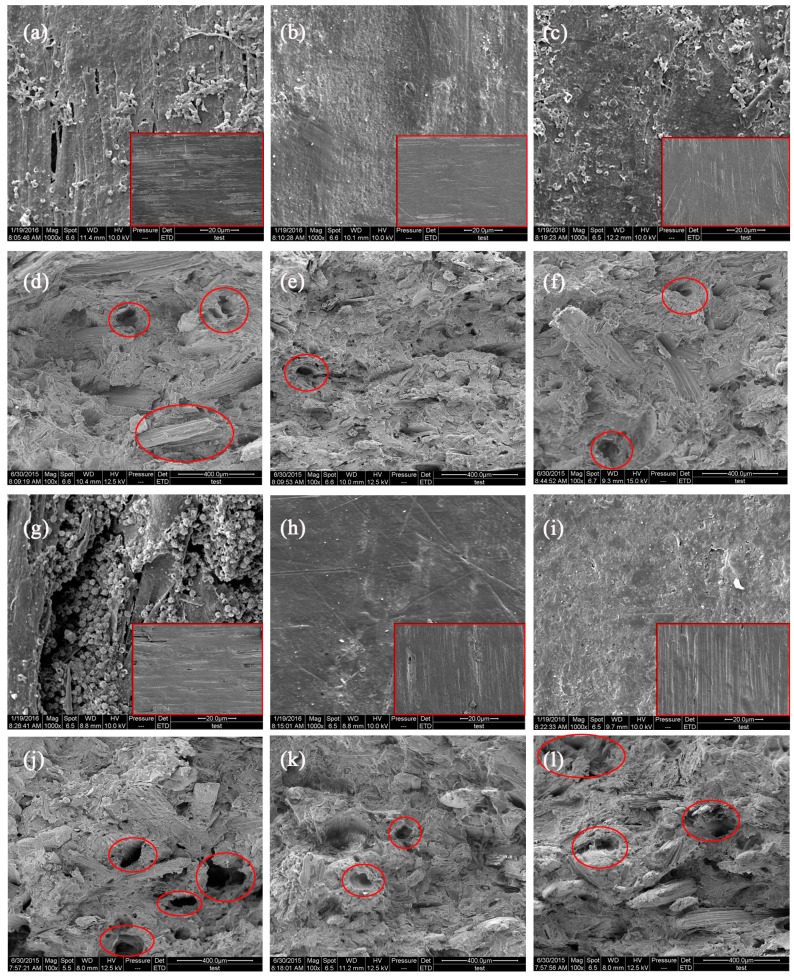
SEM images of the typical damage of white rot fungi activity on representative specimens from all CSF/HDPE formulations tested. (**a**) Surface-CSF content 50%, untreated; (**b**) Surface-CSF content 50%, ACQ 3%; (**c**) Surface-CSF content 50%, ZB 3%; (**d**) Cross section-CSF content 50%, untreated; (**e**) Cross section-CSF content 50%, ACQ 3%; (**f**) Cross section-CSF content 50%, ZB 3%; (**g**) Surface-CSF content 60%, untreated; (**h**) Surface-CSF content 60%, ACQ 3%; (**i**) Surface-CSF content 60%, ZB 3%; (**j**) Cross section-CSF content 60%, untreated; (**k**) Cross section-CSF content 60%, ACQ 3%; (**l**) Cross section-CSF content 60%, ZB 3%.

**Figure 8:**
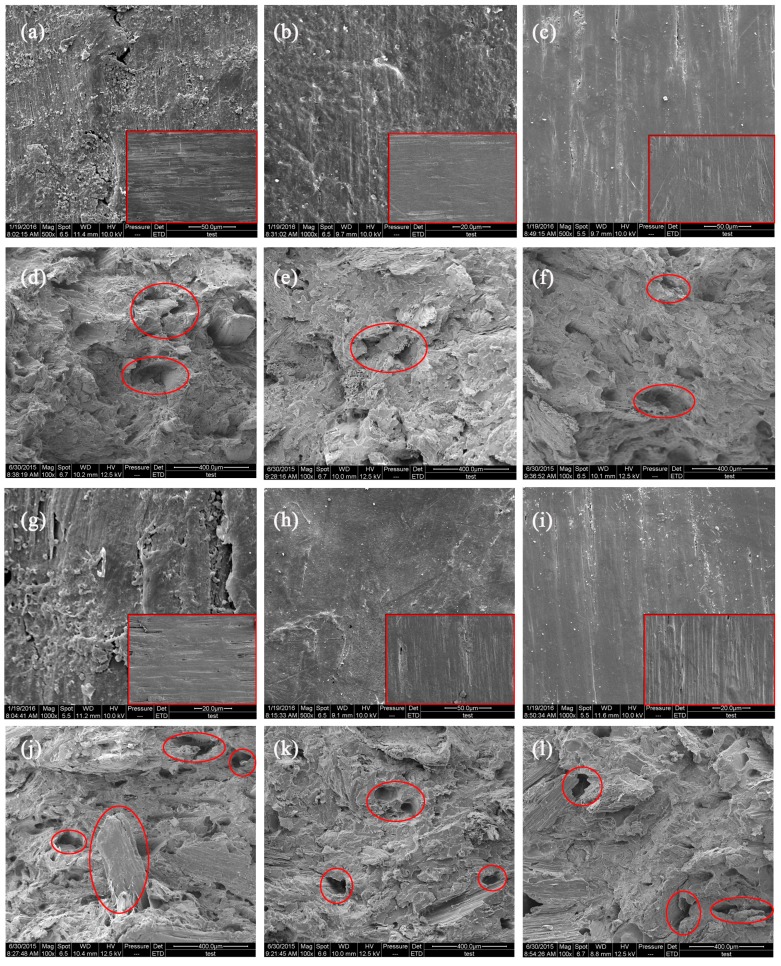
SEM images of the typical damage of brown rot fungi activity on representative specimens from all CSF/HDPE formulations tested. (**a**) Surface-CSF content 50%, untreated; (**b**) Surface-CSF content 50%, ACQ 3%; (**c**) Surface-CSF content 50%, ZB 3%; (**d**) Cross section-CSF content 50%, untreated; (**e**) Cross section-CSF content 50%, ACQ 3%; (**f**) Cross section-CSF content 50%, ZB 3%; (**g**) Surface-CSF content 60%, untreated; (**h**) Surface-CSF content 60%, ACQ 3%; (**i**) Surface-CSF content 60%, ZB 3%; (**j**) Cross section-CSF content 60%, untreated; (**k**) Cross section-CSF content 60%, ACQ 3%; (**l**) Cross section-CSF content 60%, ZB 3%.

The scanning electron microscopy images of the surface and internal microstructures of samples before and after the decay resistance test are presented in Figure 7 and Figure 8. All types of samples had smooth surfaces before the decay resistance test. Large quantities of HDPE were observed on the surface of decayed composites. The damage was limited to the material surface for the 50/50 formulations. A consequence of increasing the CSF content to 60% was that the material had more damage, with deep pits and fungi visible on the composite surface, particularly for the untreated composites (Figure 7g and Figure 8g). These results indicate that the CSF/HDPE composites lacking a preservative blocked biological agents because of the encapsulation properties of the plastic matrix alone. The observed biodegradation was less extensive in the ACQ-treated or ZB-treated CSF/HDPE composites than in the untreated controls. Additionally, the ACQ-treated materials had minimal surface damage with very little mycelial growth (Figure 7b,h and Figure 8b,h). Meanwhile, compared with untreated composites, the cross sections of ACQ-treated composites exhibited a continuous matrix and CSF filler, but no mycelia growth was observed, indicating that degradation mainly occurred on the surface of the composites during the experiment. (Figure 7e,k and Figure 8e,k). However, the ZB-treated samples with the same CSF content were covered with mycelia and voids, indicating that the ZB treatment had little effect on decay resistance (Figure 7c,i and Figure 8c,i). Some small cracks of individual CSF components were observed in the cross sections (Figure 7f,l and Figure 8f,l). Fabiyi [9] also proved that the degree of fungal degradation was linked with voids on the remaining composites. Regarding the effect of different fungi on the anti-biological properties of composites in the same formulations, *P. sanguineus* can damage the surface of composite materials by degrading the CSF. The scanning electron microscopy results are consistent with the mass loss data presented above.

## 4. Conclusions

Our results revealed that the incorporation of ACQ or ZB by CSF/HDPE composites can significantly decrease mass losses and mold growth. This implies that these biocides may be inhibitory toward termites, mold fungi, and wood-decay fungi, even at high CSF formulations (e.g., 60%). The following are specific conclusions that are supported by the results described herein:

1. CSF/HDPE containing 3% ACQ is more resistant to termites, white rot fungi, and brown rot fungi than the untreated control composites, even at high CSF formulations (e.g., 60%). Additionally, ACQ enhances the biotic stress resistance of CSF/HDPE composites more than ZB.
2. Compared with the untreated poplar/HDPE composite, the untreated CSF/HDPE is more susceptible to mold infections and decay.
3. The CSF of the CSF/HDPE composites is susceptible to fungal attacks, with the soft rot decay causing the largest mass losses, followed by the white rot fungus, and then the brown rot fungus.
4. Manufacturing AFPCs using preservative-treated CSF may be feasible, and represents a potentially viable way to convert an agricultural waste product into value-added composites.

## Figures and Tables

**Table 1:** Blend design for the CSF/HDPE composite samples.

Code	Fiber (wt %)	HDPE (wt %)	ACQ (phc)	ZB (phc)	MAPE (wt %)	Paraffin (wt %)
A	CSF-50	47	1	-	2	1
B	CSF-50	47	2	-	2	1
C	CSF-50	47	3	-	2	1
D	CSF-60	37	1	-	2	1
E	CSF-60	37	2	-	2	1
F	CSF-60	37	3	-	2	1
G	CSF-50	47	-	1	2	1
H	CSF-50	47	-	2	2	1
I	CSF-50	47	-	3	2	1
J	CSF-60	37	-	1	2	1
K	CSF-60	37	-	2	2	1
L	CSF-60	37	-	3	2	1
M	CSF-50	47	-	-	2	1
N	CSF-60	37	-	-	2	1
O	Poplar-50	47	-	-	2	1
P	Poplar-60	37	-	-	2	1

Note: phc represents the abbreviation of per hundred compounds.

**Table 2:** Mold growth severity rating over the CSF/HDPE specimens.

Rating	Mold Coverage (%) over Materials Surface
0	No mold coverage/growth (sound specimen)
1	Range: 1–5% mold coverage (median: 3%)
2	Range: 6–20% mold coverage (median: 13%)
3	Range: 21–35% mold coverage (median: 28%)
4	Range: 36–50% mold coverage (median: 43%)
5	Range: 51–75% mold coverage (median: 63%)
6	Range: 76–100% mold coverage (median: 88%)

**Table 3:** Termite test data for HDPE composites reinforced with treated and untreated CSF.

Sample Group	Type of CSF	Preservative Level (wt %)	Air Dry Moisture Content (%)	Mass Loss (%)	Visual Rating
CSF/HDPE = 50/50	untreated	0	0.58(0.04) ^ab^	3.6(0.5) ^ef^	8.8(0.8) ^bcd^
	ACQ treated	1	0.97(0.27) ^d^	2.3(0.1) ^bc^	9.6(0.4) ^cde^
		2	0.88(0.08) ^bc^	1.4(0.1) ^cd^	9.5(0.0) ^bcde^
		3	0.83(0.11) ^cd^	1.0(0.1) ^bc^	9.6(0.1) ^bc^
	ZB treated	1	0.48(0.01) ^a^	2.8(0.4) ^ab^	9.5(0.3) ^bcde^
		2	0.49(0.11) ^a^	2.5(0.3) ^b^	9.5(0.0) ^bcde^
		3	0.40(0.03) ^a^	2.0(0.4) ^c^	9.3(0.7) ^bcd^
CSF/HDPE = 60/40	untreated	0	0.58(0.06) ^ab^	5.5(0.4) ^h^	9.0(0.8) ^bc^
	ACQ treated	1	0.94(0.11) ^cd^	3.5(0.1) ^cd^	9.8(0.3) ^de^
		2	0.86(0.12) ^bc^	3.2(0.1) ^de^	9.6(0.2) ^cde^
		3	1.65(0.56) ^e^	2.5(0.4) ^cd^	9.8(0.3) ^de^
	ZB treated	1	0.58(0.02) ^ab^	5.3(0.2) ^ef^	9.4(0.6) ^bcde^
		2	0.63(0.02) ^abc^	4.7(0.3) ^gh^	9.4(0.2) ^b^
		3	0.63(0.05) ^abc^	3.6(0.5) ^h^	9.3(0.8) ^bcd^
Wood control	Poplar *	-	11.12(0.38) ^f^	100.0(0.0) ^i^	0.0(0.0) ^a^

Note: * poplar used as reference material. ^abcde^ Means (standard deviation) followed by different superscript letters within the same column are significantly different at the level of *p* ≤ 0.05.

**Table 4:** Mold test data for HDPE composites reinforced with treated and untreated CSF.

Sample Group	Type of Material	Preservative Level (wt %)	Time for the First Occurrence of Mold Attack	Median Mold Growth after the Test Completed (%)
CSF/HDPE = 50/50	ACQ-treated CSF	1	No mold growth	0
		2	No mold growth	0
		3	No mold growth	0
	ZB-treated CSF	1	Day 9	7
		2	Day 9	5.13
		3	Day 10	3.85
	Untreated CSF	-	Day 9	12.4
Wood/HDPE = 50/50	Untreated poplar	-	Day 10	5
CSF/HDPE = 60/40	ACQ-treated CSF	1	No mold growth	0
		2	No mold growth	0
		3	No mold growth	0
	ZB-treated CSF	1	Day 9	20.5
		2	Day 9	13.5
		3	Day 10	11.25
	Untreated CSF	-	Day 9	24
Wood/HDPE = 60/40	Untreated poplar	-	Day 11	6.4
Wood control 1	Pure poplar ^a^	-	Day 9	69.25
Wood control 2	Pure rubber ^b^	-	Day 2	88

Note: ^a^ poplar and ^b^ rubber used as reference materials.
